# Important considerations when assessing the effect of essential fatty acids on cognitive performance

**DOI:** 10.1186/s12937-020-00619-8

**Published:** 2020-09-11

**Authors:** Aayush Visaria, David Lo, Pranay Maniar

**Affiliations:** 1 Department of Medicine, Rutgers New Jersey Medical School, 185 South Orange Ave, Newark, NJ 07103 USA; 2 North American Disease Intervention, Rutgers University, New Brunswick, NJ USA; 3 New Jersey Institute of Technology, Newark, NJ USA

**Keywords:** Fatty acids, Nutrition, Cognitive impairment, Elderly

## Abstract

Over the past decade, there have been many studies determining the effect of dietary ω-3 and ω-6 fatty acids intake on cognitive performance; however, they have largely been inconsistent in their conclusions. In this letter, we provide context to the article by Dong et al., titled “*Association of dietary ω-3 and ω-6 fatty acids intake with cognitive performance in older adults: National Health and nutrition examination Survey (NHANES) 2011–2014” and* provide methodological considerations with regards to covariate measurement and inclusion that can be generalized to future cross-sectional studies. In particular, delineating 1) the type and source of fatty acid, in context of an individual’s overall dietary patterns, 2) sociobehavioral risk factors and physical & mental comorbidities, 3) and daily cognitive activity are important to adequately control for covariates.

To the Editor:

Nutrition is at the forefront of preventive medicine, with ever-increasing evidence of the benefit of different nutrients and their ability to prevent, mitigate the progression of, and even reverse chronic diseases or processes. One such process is cognitive decline. We read with great pleasure the article by Dong et al., titled “*Association of dietary ω-3 and ω-6 fatty acids intake with cognitive performance in older adults: National Health and nutrition examination Survey (NHANES) 2011–2014*” which found that increased dietary ω-3 and ω-6 fatty acids were inversely associated with low cognitive performance in people aged 60 and older [1]. We applaud the authors’ efforts to comprehensively control for potential confounders such as physical activity and comorbidity burden; however, we would like to reiterate and bring forth key covariates and methodological considerations that may be necessary to determine the independent effect of *ω-3 and ω-6* fatty acids on cognitive performance.

While the authors have appropriately controlled for demographic, socioeconomic, physical activity, and cardiometabolic comorbidity factors, there are several other covariates that need to be accounted for, including 1) vitamin and mineral consumption (as authors have mentioned, vitamin Bs [2], vitamin D [3], folate [4], among others, have all shown protective effects on cognitive function), 2) other dietary consumption (protein intake [5] has been shown to be protective whereas saturated fats and refined sugars [6] have been associated with cognitive decline), 3) source of fatty acid (plant-based vs. animal-based [7]), 4) mental health status (e.g. depression [8] using the nine-item Patient Health Questionnaire), 5) other non-cardiometabolic comorbidities such as liver disease, pre-diabetes, and gastrointestinal disorders [9] that alter the gut microbiome, 6) behavioral factors such as smoking status [10], 7) day-to-day activities (e.g. retired vs. stay at home vs. workplace as well as type of work), and 8) variability of 24-h dietary recall from first interview to second interview to get an idea of an individual’s dietary consistency. It is understandable that some of these covariates were not included as analysis is constrained by the quality and depth of the National Health and Nutrition Examination Survey (NHANES) datasets. However, cognitive performance is a highly fluid and complex measurement that is affected by physical, mental, and emotional aspects of one’s life. The authors have incorporated several of the above covariates in their other studies using NHANES [5, 8] and thus are available and should be included in future studies.

An increasingly well-accepted notion of nutrition is that the whole is greater than the sum of its parts. This is important when assessing the effect of essential fatty acids because the source of the fatty acid can impact how it is metabolized in the body and its resulting bioavailability. The *ω-3* and *ω-6* fatty acids, along with the endogenously produced *ω-9* fatty acids, share enzymes and thus can be affected by one another. This seesaw type of relationship among these fatty acids can impact their association with cognitive performance. Additionally, dietary fatty acids may not directly be associated with serum fatty acid levels as metabolism is different from individual to individual; this may explain the insignificant association of the *ω-6: ω-3* ratio. Refined sugars and saturated fats, as noted by the authors, are positively associated with low cognitive performance. Whether the negative effects of these macronutrients outweigh the positive effects of *ω*-acids is unknown. It would be worthwhile then to include all aspects of one’s diet into the analysis. Vascular-related dementia, a major cause of cognitive impairment, has risk factors similar to cardiovascular disease; this would suggest that all the risk factors that have been established in cardiovascular disease literature are potential confounders, further complicating the analysis.

An important limitation of the NHANES surveys is that many of the comorbidities and dietary information is based on self-recall. Whether this self-recall is affected by one’s cognitive function (e.g. memory) is unknown, but we can speculate that those with cognitive impairment may not recall as accurately as others, leading to potential bias.

Ultimately, we believe that a truly interpretable cross-sectional study would have 1) increased cognitive performance measurements to incorporate different domains, 2) dietary information based on real-time input into an online platform, 3) measurement of covariates as mentioned, and 4) stratified analyses by vegetarian vs. non-vegetarian diet and by cardiometabolic status (e.g. metabolic syndrome).

Overall, we believe the authors have made a strong case for the inverse association between ω-3 and ω-6 fatty acids and low cognitive performance in older adults. Further studies delineating the various intricacies of fatty acid nutrition and metabolism and complex relationship with confounders are needed to help reach a consensus.

## Data Availability

Not applicable.
